# Statistical significance and its critics: practicing damaging science, or damaging scientific practice?

**DOI:** 10.1007/s11229-022-03692-0

**Published:** 2022-05-12

**Authors:** Deborah G. Mayo, David Hand

**Affiliations:** 1 Virginia Tech, Blacksburg, USA; 2 Imperial College London, London, UK

**Keywords:** Data-dredging, Error probabilities, Fisher, Neyman and Pearson, P-values, Statistical significance tests

## Abstract

While the common procedure of statistical significance testing and its accompanying concept of p-values have long been surrounded by controversy, renewed concern has been triggered by the replication crisis in science. Many blame statistical significance tests themselves, and some regard them as sufficiently damaging to scientific practice as to warrant being abandoned. We take a contrary position, arguing that the central criticisms arise from misunderstanding and misusing the statistical tools, and that in fact the purported remedies themselves risk damaging science. We argue that banning the use of p-value thresholds in interpreting data does not diminish but rather exacerbates data-dredging and biasing selection effects. If an account cannot specify outcomes that will not be allowed to count as evidence for a claim—if all thresholds are abandoned—then there is no test of that claim. The contributions of this paper are: To explain the rival statistical philosophies underlying the ongoing controversy; To elucidate and reinterpret statistical significance tests, and explain how this reinterpretation ameliorates common misuses and misinterpretations; To argue why recent recommendations to replace, abandon, or retire statistical significance undermine a central function of statistics in science: to test whether observed patterns in the data are genuine or due to background variability.

## Introduction and background

While the common procedure of statistical significance testing and its accompanying concept of p-values have long been surrounded by controversy, renewed concern has been triggered by the so-called replication crisis in some scientific fields. In those fields, many results that had been found statistically significant are not found to be so (or have smaller effect sizes) when an independent group tries to replicate them. This has led many to blame the statistical significance tests themselves, and some view the use of p-value thresholds as sufficiently damaging to scientific practice as to warrant being abandoned. We take a contrary position, arguing that the central criticisms arise from misunderstanding and misusing the statistical tools, and that in fact the purported remedies themselves risk damaging science. In our view, if an account cannot specify outcomes that will not be allowed to count as evidence for a claim—if all thresholds are abandoned—then there is no test of that claim.

In this paper we propose to explain why some of today’s attempts to fix statistical practice are actually jeopardizing reliability and integrity. Even where critics of statistical significance tests are mainly objecting to misuses and misinterpretations, recommended fixes often grow out of controversial underlying conceptions about the nature and role of statistics in science. Philosophers of science are immersed in many areas beset with uncertainty and statistical models and methods are pervasive. Getting a handle on the current controversies in statistical foundations is important to the success of such projects, hence this special issue on *Recent Issues in Philosophy of Statistics: Evidence, Testing, and Applications.* The controversy has generated a huge and unwieldy literature over many years. We will use the recent and ongoing controversy involving the American Statistical Association (ASA) as a vehicle for us to highlight the central issues and zero in on the current state of play in the debates.

To outline our goals, we begin with some background. In 2016 the American Statistical Association (ASA) issued a *Statement on P-Values and Statistical Significance* intended to highlight classic misinterpretations and abuses (Wasserstein & Lazar, 2016, hereafter, 2016 ASA Statement). The six principles^1^ it offers are a mix of statements of familiar properties of p-values, well-known misunderstandings, and guidance on the correct usage. But in March 2019, an Executive Director’s editorial introducing 43 papers in a special issue of *The American Statistician* (“Statistical Inference in the 21st Century: A world beyond ‘*p* < 0.05’”), declared that: “[the 2016 ASA Statement] stopped just short of recommending that declarations of ‘statistical significance’ be abandoned” (Wasserstein et al., 2019, p. 2, hereafter, WSL 2019). They announce: “We take that step here….[I]t is time to stop using the term ‘statistically significant’ entirely. …” (WSL 2019, p. 2). They do not propose to ban p-values, but they do propose banning the phrases “statistical significance/statistically significant” and hold that “whether a *p*-value passes any arbitrary threshold should not be considered at all when deciding which results to present or highlight” (ibid.). We may call this the “no threshold view”.

Our main focus is on the no threshold view (WSL 2019) as well as other criticisms of statistical significance. (Our reference to WSL 2019 refers only to the opening sections, not the summaries of papers in the issue.) We shall also consider accounts that retain p-value thresholds, but call for appraising or using them in Bayesian computations. Since all of these positions vary and even disagree with one another, we will identify the relevant theses as we proceed.

The process that professional societies use to argue against a methodology that is widespread in science matters a good deal when it comes to the very goal they are supposed to be championing—trust in science. To claim, as WSL 2019 does, that a declaration of statistical significance is the “antithesis of thoughtfulness” (p. 4) is misleading and uncharitable. We will argue that abandoning a concept or tool which can be of great value when it is used properly merely because it has been misused in the past is itself the apotheosis of thoughtlessness. Further, we argue that a trustworthy critique of statistical significance tests should specifically consider the responses of testers themselves.

Concerned that WSL 2019 might be taken as a continuation of the 2016 ASA Statement, in 2019 the Board of the ASA appointed a President’s Task Force on Statistical Significance and Replicability. It was put in the odd position of needing to “address concerns that [the Executive Director’s editorial, WSL 2019] might be mistakenly interpreted as official ASA policy” (Benjamini et al., 2021). Their recently published report concludes: “P-values and significance testing properly applied and interpreted, are important tools that should not be abandoned” (ibid.). We concur, but will go far beyond their one-page report to give detailed arguments for advancing this position. The goals of our paper are:

- To explain the key issues in the ongoing controversy surrounding statistical significance tests;
- To reinterpret statistical significance tests, and the use of p-values, and explain how this reinterpretation ameliorates common misuses that underlie criticisms of these methods;
- To show that underlying many criticisms of statistical significance tests, and especially proposed alternatives, are often controversial philosophical presuppositions about statistical evidence and inference;
- To argue that recommendations to replace, abandon, or retire statistical significance tests are damaging to scientific practice.

Section 2 sets out the main features of statistical significance tests, emphasizing aspects that are routinely misunderstood, especially by their critics. In Sects. 3 and 4 we will flesh out, and respond to, what seem to be the strongest arguments in support of the view that current uses of statistical significance tests are damaging to science. Section 3 explores five key mistaken interpretations of p-values, how these can lead to damaging science, and how to avoid them. In Sect. 4 we discuss and respond to central criticisms of p-values that arise from presupposing alternative philosophies of evidence and inference. In Sect. 5 we argue that calls to replace, abandon, or retire statistical significance tests are damaging to scientific practice. We argue that the “no threshold” view does not diminish but rather exacerbates data-dredging and biasing selection effects (Sect. 5.1), and undermines a central function of statistics in science: to test whether observed patterns in the data can be explained by chance variation or not (Sect. 5.2). Section 5.3 shows why specific recommendations to retire, replace, or abandon statistical significance yield unsatisfactory tools for answering the significance tester’s question. Finally, in Sect. 6 we pull together the main threads of the discussion, and consider some implications for evaluating statistical methods with integrity.

## P-values and statistical significance

Statistical significance tests are part of a rich piecemeal set of tools intended to assess and control the probabilities of misleading interpretations of data—often called *error probabilities*. Because of this role, the tools may be described as *error statistical*, a much more apt and inclusive term than “frequentist”. The set includes simple Fisherian tests, Neyman–Pearson (N–P) formulations of hypothesis tests, confidence intervals, randomization, resampling methods and much else. Statistical significance tests address the particular error of mistaking a feature of the data that has arisen because of random variability for a genuine underlying effect. As Fisher (1956, p. 79) wrote: “[tests of significance] are constantly in use to distinguish real effects of importance to a research programme from such apparent effects as might have appeared in consequence of errors of random sampling, or of uncontrolled variability, of any sort, in the physical or biological material under examination”. As Yoav Benjamini puts it, significance tests are our “first line of defense against being fooled by randomness” (2016, p. 1).

### Elements of statistical significance tests

A statistical hypothesis *H* is a claim about some aspect of the process that might have generated the data. For example, a common statistical hypothesis, *H*_0_, has the form that an experimental intervention has “no effect" or produces "no difference". The ubiquity of this “no effect” hypothesis explains why Fisher's term *null hypothesis* is often used. A more apt term is Neyman and Pearson's *test hypothesis*, because we do not want to limit ourselves to “no effect” since it is often quite artificial. Still, we use “null” for brevity. For example, a general form of test hypothesis asserts that some characteristic of the process that generated the data has a value δ that is less than some value of interest δ'. In discussing a Covid-19 trial run by AstraZeneca (AZ), statistician Stephen Senn notes that “the null hypothesis that is being tested is not that the vaccine has no efficacy but that its efficacy does not exceed 30%” (Senn, 2020). In general:

- The immediate objective is to test the conformity of the particular data under analysis with *H*_0_ in some respect to be specified. To do this we find a function *d* = *d*(***x***) of the data, to be called the *test statistic*, such that
  - the larger the value of* d *the more inconsistent are the data with *H*_0_;
  - the corresponding random variable *D*  = *d*(***X***) has a (numerically) known probability distribution when *H*_0_ is true. (Mayo & Cox, 2006, p. 81, T replaced with D, *y* with *x*)

The observed *significance level* or p-*value* associated with *d*(***x***_0_) is the probability of getting at least as extreme a value as *d*(***x***_0_) computed under *H*_0_, where ***x***_0_ is the observed sample.

$${\text{p - value }} = {\text{ Pr}}\left( {d{(\varvec{X}}) \ge d({\varvec{x}}_{0} );H_{0} } \right).$$

In words, the p-value is the probability that the test would have produced a result differing from *H*_0_ at least as much as the one observed, if *H*_0_ is the case.

Note that we compute the probability of (*d*(***X***) ≥ *d*(***x***_0_)) under the assumption *H*_0_, stressing the inequality. We cannot just look at the probability of the particular observation, Pr(*d*(***X***) = *d*(***x***_0_); *H*_0_) because any continuous observation is going to be improbable under *H*_0_. Yet it is very common to find formulations of statistical significance tests that describe tests as declaring a result ***x***_0_ statistically significant if ***x***_0_ is not likely to occur assuming the null hypothesis is true. This is wrong. We would not declare a result statistically significant simply because it is improbable under *H*_0_.^2^ If we did, a test would very probably declare results statistically significant erroneously, violating the error probability guarantees.

The reasoning, Cox and Hinkley (1974, p. 66) explain, is this:

Suppose that we were to accept the available data as evidence against *H*_0_. Then we would be bound to accept all data with a larger value of [*d*] as even stronger evidence. Hence *p*_obs_ [the observed p-value] is the probability that we would mistakenly declare there to be evidence against *H*_0_, were we to regard the data under analysis as just decisive against *H*_0_.

Note that what is being accepted or not are claims that the data provide evidence against *H*_0_—not claims to accept or reject *H*_0_ itself. To “accept” or “reject” a hypothesis is really just a shorthand for these claims about evidence, at least in contexts of scientific inference.

We recommend this reading in relation to a given test T: A p-value is the probability test T would have given rise to a result more incompatible with *H*_0_ than *d*(***x***_0_) is, were the results due to background or chance variability, as described in *H*_0_. It is a counterfactual claim. The error probability is accorded to the test *procedure*, not to the observed data. In other words, we understand the capacities of the test by considering how it would behave with other data generated under *H*_0_.

### Neyman and Pearson (N–P) tests

Neyman and Pearson (N–P) tests introduce the alternative statistical hypothesis *H*_1_ where *H*_0_ and *H*_1_ are typically assertions about parameters within a model. For example, *H*_0_ might assert the vaccine efficacy δ is less than or equal to 30%, and *H*_1_ assert that δ is greater than 30%.^3^ The N–P test prespecifies a p-value at or beyond which *H*_0_ is rejected—thereby controlling the *Type 1 error* probability, the probability of erroneously rejecting *H*_0_. Introducing the alternative hypothesis *H*_1_ also allows control of the *Type 2 error*: failing to find statistical significance when there is a genuine discrepancy δ' from *H*_0_. The complement is the test’s *power* to detect δ'. Having fixed a threshold for the maximal Type 1 error probability, N–P sought tests to minimize the Type 2 error probability or, equivalently, maximize the test’s power to detect alternatives. A sensible test must have a higher probability of rejecting *H*_0_ when *H*_1_ is true than when *H*_0_ is true.

Their success at finding tests with good if not optimal error probabilities established a new pattern for appraising statistical methods that continues to be important in statistics and machine learning. However, it also encouraged the perception that hypothesis tests, rather than being tools for inference, are rules for deciding if a hypothesis should be rejected or accepted according to how well tests would perform in some long run of applications. Call this the *performance* construal of N–P tests. But, it is not the only, or the best, way to view them. The truth is that N–P were trying to provide a more rigorous framework for Fisherian tests. Wanting to distinguish their account from inductive logics of the day,^4^ they viewed the “decision” to interpret data as a kind of action. Instead of inductive inference, Neyman (1957) spoke of *inductive behavior*.

### Performance vs probativeness

David Cox usefully connects Fisherian and N–P tests by considering the p-value as a function to be computed under alternatives *H*_1_ as well as under *H*_0_.

In the Neyman–Pearson theory of tests, the sensitivity of a test is assessed by the notion of *power*, defined as the probability of reaching a preset level of significance considered for various alternative hypotheses. In the approach adopted here the assessment is via the distribution of the random variable *P*, … for various alternatives. (Cox, 2006, p. 25)

In other words, the p-value can refer both to the random variable P and also to its particular value. Once the p-value is known, one can compute Pr(*d*(***X***) ≥ *d*(***x***_0_); *H*′) for various alternatives *H*′ of interest. Cox stresses that the calibration of tests in terms of how often we would erroneously interpret data is solely to convey the meaning of terms. We agree. In scientific contexts, the relevance of the overall performance of the tool is not merely to ensure we will avoid erroneously interpreting data in the long run. It is informative for understanding *the capacities* of the tools that apply in the case at hand. (For a full development of this philosophy of statistics, see Mayo, 1996, 2018; Mayo & Spanos, 2011.)

Minimally if a tool is incapable of having uncovered the falsity of, or flaws in, a claim, then finding none fails to provide evidence for that claim. This gives a minimal requirement for a good test or evidence. This applies whether we are dealing with a formal test, a method of estimation, or one of prediction:

*Minimal requirement for evidence:* We have evidence for *H* only if *H* passes a test that probably would have found evidence of flaws in *H*, if they are present.

This probability is the strength or *severity* of the test *H* has passed with the data being considered. We also hold the converse of this claim: Data ***x*** provide evidence for a claim *H* to the extent that *H* has passed a severe test with ***x***. In this way, a test’s error probabilities are used to evaluate how well or poorly probed claims are. We dub this the *probativeness* use of error probabilities. Good long-run performance is a necessary but not a sufficient condition for a test to calibrate probativeness. In our view, a probative test will generally involve combining several subsidiary tests, deliberately designed to unearth different flaws.

The reasoning supplies a statistical *falsification* of *H*_0_*.* If the test would produce even larger differences than *d*(***x***_0_) fairly frequently assuming *H*_0_ to be true (that is, the p-value is not small), then there is little evidence of incompatibility with *H*_0_. We would then have no reason to suppose *H*_0_ to be a poor explanation of the data. By contrast, if the p-value is small (and what should be regarded as “small” will depend on the research aims and context), then with high probability the test would have produced a smaller difference than we observed, were *H*_0_ in fact adequate. To put this in Popperian terms, if *H*_0_ very probably would have “survived” the test, if true, and yet the test yields a result discordant with *H*_0_, then it gives an indication of *the denial of H*_0_. In speaking of statistical falsification, Popper notes that although extremely rare events may occur:

such occurrences would not be physical effects, because, on account of their immense improbability, *they are not reproducible* at *will* … If, however, we find *reproducible* deviations from a macro effect . . . deduced from a probability estimate …. then we must assume that the probability estimate is *falsified*. (Popper, 1959, p. 203)

## Damaging misrepresentations and how to avoid them

### Statistical significance is erroneously taken to mean scientifically important

A central criticism is that calling something “significant” in ordinary English connotes importance. Placing “statistical” before “significance” is intended to have the diminutive effect to avoid misinterpretation. It is saying merely that the observed effect or difference is not readily explained by random or chance variability. “Statistical significance was never meant to imply scientific importance”, WSL 2019 correctly observes (p. 2). To the authors, the confusion between the statistical and ordinary language meanings of the word is itself grounds to avoid it. A term other than significance might well be preferred, for example “is statistically distinguishable from”, or “is statistically inconsistent with”, random error, at the given level. But any words used are going to be open to misinterpretation (as with “probable”, “likely”, and “confidence”).

Particularly pernicious is the practice of inferring a substantive research claim from a single statistically significant result. It is this fallacy that is at the heart of why statistical significance tests are often blamed for a high rate of failed replication:

[T]he high rate of nonreplication of research discoveries is a consequence of the convenient, yet ill-founded strategy of claiming conclusive research findings solely on the basis of a single study assessed by formal statistical significance, typically for a *p*-value less than 0.05. … (Ioannidis, 2005, p. 0696)

In fact, even to obtain grounds for a genuine statistical effect requires more than the isolated p-values that Ioannidis describes. From the start, R.A. Fisher emphasized:

[W]e need, not an isolated record, but a reliable method of procedure. In relation to the test of significance, we may say that a phenomenon is experimentally demonstrable when we know how to conduct an experiment which will rarely fail to give us a statistically significant result. (Fisher, 1935a, p. 14)

In fields beset by nonreplication, many researchers have not heeded their chief protagonist back in the 1970s, who warns “[T]he almost universal reliance on merely refuting the null hypothesis as the standard method for corroborating substantive theories in the soft areas... is basically unsound, poor scientific strategy...” (Meehl, 1978, p. 817).

Even with respect to inferring the existence of a genuine effect, the p-value should not be used rigidly. In good practice, conventional choices, such as 0.01, 0.025, 0.05, are set when they are found to correspond to useful Type 1 and 2 error probabilities for a given field and stage of research. We should not confuse prespecifying minimal thresholds in each test, which we uphold, with fixing a value to use routinely (which we would not). Granted, conventional choices for thresholds may often be used thoughtlessly. Neyman and Pearson advised that tests be used with “discretion and understanding” (1928, p. 58), claiming “it is doubtful whether the knowledge that *P*_*z*_ [the p-value associated with test statistic *z*] was really 0.03 (or 0.06) rather than 0.05,... would in fact ever modify our judgment... regarding the origin of a single sample (ibid., p. 27). Even Neyman’s first student, Erich Lehmann, who was responsible for developing the influential behavioral-decision formulation of tests, recommends that post-data, researchers report the observed p-value, which “gives an idea of how strongly the data contradict the hypothesis. It also enables others to reach a verdict based on the significance level of their choice” (Lehmann & Romano, 2005, pp. 63–64). We agree, but to make this choice wisely requires considering the effect sizes (or population discrepancies) indicated by achieving a given significance level.

### The p-value does not measure the size of a population effect

Damaging misunderstandings occur from supposing a small p-value indicates a large magnitude of a population effect, while supposing a lack of statistical significance means a small population effect size. A p-value is a probability, not a measure in units of the hypothesized parameter. At most it is to be taken as a report of evidence of the existence of an inconsistency with the null hypothesis. Determining how large an inconsistency is indicated is a distinct step. However, error statistical methods do provide ways to carry out this step. A simple way, using only p-values, is to consider, not just a single null hypothesis, but several discrepancies from that null hypothesis.

Suppose we are testing the mean μ of a Normal distribution: *H*_0_: μ ≤ μ_0_ versus *H*_1_: μ > μ_0_, with a random sample of size n. This is a 1-sided test, because only positive discrepancies from μ_0_ are being probed. We observe, $\overline{\text{x}},$ the value of the sample mean, $\overline{\text{X}},$ and compute how much it differs from μ_0_ in standard error (SE) units. That is, the test statistic *d* is $(\overline{\text{X}} - \mu_{0})/{\text{SE}}.$ If the p-value is small, it is indicative of *some* discrepancy from *H*_0_, but we are concerned about the magnitude of that discrepancy. That is, we are interested in alternatives of the form μ_1_ = μ_0_ + γ (γ being a discrepancy parameter). A general error statistical testing principle is:

*Error statistical testing principle (i)* (for avoiding a magnitude error): If there is a fairly high probability that *d* would have been larger than observed (a probability > .5), even if μ is no greater than μ_1_, then *d* is a poor indication that μ > μ_1_, where μ_1_ = μ_0_ + γ (with γ > 0).

There is good evidence that μ > μ_1_ if (and only if) this probability is low.

For a toy example, let *H*_0_: μ ≤ 0 versus *H*_1_: μ > 0, and let the SE equal 1. The 2-SE cut-off giving a p-value of approximately 0.025 is 2 (0 + 2SE). If *d* (which in this case is just $\overline{\text{X}}$), is 2 or greater, the result is statistically significant at level 0.025. Suppose *d* is 2. A useful benchmark for a poorly warranted discrepancy is to consider a μ_1_ greater than $\overline{\text{X}},$ e.g., $\it \overline{\text{X}}+{\text{1SE}}\;({\text{i.e.,\,3}}).$ The hypothesis μ > 3 will be poorly warranted because the probability that *d* is even larger than 2, under the assumption that μ = 3 is fairly high 0.84.^5^ By reporting various benchmarks, tests can avoid magnitude errors in interpreting p-values. (See Mayo, 2018; Mayo & Spanos, 2006, 2011.)

One can arrive in much the same place using confidence intervals developed by Neyman (1937) to estimate a range of values of a parameter. Like tests, they can be 1 or 2-sided. The (2 SE) confidence interval estimation *rule* is to estimate the mean μ of a Normal distribution as the sample mean ${\overline{\text{X}}}$ plus or minus 2 SE, i.e., $\mu = (\overline{\text{X}} \pm 2\text{SE}).$ Applications of this method result in covering the true μ, whatever it is, around 95% of the time. (Note the 0.025 is doubled because both sides are considered.) The 2-sided interval in our example with $\overline{\text{X}} = 2$ is [0 < μ < 4]. There is a direct duality between tests and confidence intervals: a confidence interval (CI) at level 1– c consists of parameter values that are not statistically significantly different from the data at significance level c. One could obtain the lower (1-sided) CI bound (at level 1– c) by asking: what parameter value is the data statistically significantly greater than at level c?

We endorse the common practice of reporting a CI along with a p-value. The trouble is that merely reporting a confidence interval does not distinguish the warrant associated with hypotheses about different points in the interval. So we think that CI advocates would benefit from considering several different thresholds for confidence levels, not just the standard 0.95.^6^

### A statistically insignificant result (a non-small p-value) is not evidence for *H*_0_

A well-known fallacy is to take the failure to find evidence against a claim as evidence for it. As the title of one influential paper calls out: “Absence of evidence is not evidence of absence” (Altman & Bland, 1995). After all, a test may have little capability of issuing a statistically significant result, even if meaningful effects were to exist. Amrhein et al. (2019), in an editorial in *Nature* heralding WSL 2019, find this fallacy sufficiently damning to “retire” the concept of statistical significance. The premise for their argument is that “a statistically non-significant result does not ‘prove’ the null hypothesis (the hypothesis that there is no difference between groups or no effect of a treatment)” (p. 305).

The remedy is not to retire the tool but rather explain why it is fallacious—and error statistical requirements enable just that. A rule that allowed inferring, from a statistically insignificant result, that *H*_0_ is proved, or even well warranted, would have high Type 2 error probabilities. This is especially so in their example (with its point null and composite alternatives).^7^ We consider Amrhein et al. (2019) further in Sect. 5.3.

Granted, formulating tests as a binary classification “reject *H*” and “accept *H*” is responsible for considerable confusion, but for Neyman the term “acceptance” was merely shorthand: “The phrase ‘do not reject *H*’ is longish and cumbersome... My own preferred substitute for ‘do not reject *H*’ is ‘no evidence against *H* is found’” (Neyman, 1976, p. 749). N-P developed power, and power analysis, to block the fallacy of non-significance. Interestingly, Neyman criticizes Fisher for occasionally moving from a large p-value to inferring the null hypothesis as: “much too automatic [because]... large values of *P* may be obtained when the hypothesis tested is false to an important degree” (1957, p. 13). Thus, a researcher needs to specify the test so that meaningful effects have a good chance of triggering significance at the level chosen (typically by ensuring the sample size is large enough). A companion principle is:

*Error statistical testing principle (ii)* (for avoiding fallacies of non-significance): If there is a low probability (i.e., less than .5) that *d* would have been larger than observed, even if μ is as great as μ_1_, then *d* is not a good indication that μ < μ_1_, where μ_1_ = μ_0_ + γ (with γ > 0).

The severity with which μ < μ_1_ has passed is less than 0.5. The data begin to indicate that μ < μ_1_ (for a given μ < μ_1_) only to the extent that this probability is high, e.g., 0.8, 0.9, 0.95, 0.99, etc. Rather than set a single cut-off, with data *d* in hand, we recommend reporting the severity associated with different assertions of form μ < μ_1_, varying the values of μ_1_.

### P-values are uninterpretable if there has been p-hacking, data-dredging or a variety of biasing selection effects

The most damaging mistake, and one that is surely the main culprit behind many failures to replicate, is selective reporting and exploiting a variety of selection effects (e.g., Gelman & Loken, 2014; Ioannidis, 2005; Simmons et al., 2011). These gambits—often placed these days under the label of “p-hacking”—increase a test’s Type 1 error probability. A report of a small p-value suggests the results are very difficult to achieve under the assumption of background variability alone. However, impressive-looking results are easy to achieve if one can ignore unwelcome results, alter what is being tested post-data, or exploit a variety of sources of researcher flexibility (e.g., exclusion criteria, data dredging, stopping rules). To the extent that the calculation does not take account of the selection process, then the p-value has been miscalculated. It is not a genuine p-value.

Suppose, for example, that N sets of differences are examined, and all but the one that appears large enough to test are erased. With a single hypothesis the possible results are the values of a single *d*(***X***); now the possible results are all the hypotheses that might be found to reach a small significance level, say 0.05. The probability of finding at least one such *nominally* statistically significant difference *d*_*i*_(***x***_0_) out of N (in either direction), even though all N null hypotheses are true, will be much greater than 0.05. For example, with 20 independent samples from populations with true differences of 0, the probability of finding at least one statistically significant difference at the 0.05 level is 1 − Pr(all 20 are non-significant). This is 1 − (0.95)^20^ = 1 − 0.36 or 0.64.^8^

Perhaps the oldest method for dealing with this is the Bonferroni correction, dating from the first half of the twentieth century. This requires setting the significance level to p/N, with N the number of factors or, in medical testing, endpoints. There are many more sophisticated approaches, and this continues to be a research area in its own right. One example of a less conservative approach, developed by Benjamini and Hochberg (1995), is based on the *false-discovery rate* (FDR): the expected proportion of the N hypotheses tested that are falsely rejected. Often, selection effects have been so tortuous that it is not possible to ascertain a correct p-value but, even then, it is poor practice not to report that selection has occurred. (See 5.1.)

### A p-value can be invalidated by violations of the underlying model

As with other statistical methods, statistical significance tests depend on the assumptions underlying the model. Although assumptions of a statistical model must be met adequately in order for the p-value to operate in a test of *H*_0_, it is not required that we have an exactly correct model—whatever that would mean. A model need not be true in order to learn true things by means of it. It is required only that the error probabilities of the test are approximately related to the actual ones. (See Box, 1983; Mayo, 2018; Mayo & Spanos, 2004; Spanos, 2007, 2018.)

Some critics of statistical significance tests erroneously suppose that imperfect statistical models create an insurmountable obstacle to the usefulness of tests. David Trafimow, co-editor of the journal *Basic and Applied Social Psychology*, which has banned the use of p-values altogether, remarked in a recent National Institute of Statistical Science (NISS) debate (October 15, 2020): “it’s tantamount to impossible that the model is correct, … And so what you’re in essence doing then, is you’re using the P-value to index evidence against a model that is already known to be wrong. … so there’s no point in indexing evidence against it” (NISS, 2020, 08:44). But the p-value is not indexing evidence against the underlying model: the p-value is not tracking model violations. Statistical significance tests are, by definition, formulated in terms of a particular test statistic. The chosen test statistic measures discordance of a particular kind between the data and *H*_0_, not more general discrepancies between the data and the hypothesis or the data and the model. To the extent that underlying model assumptions are mistaken, the p-value could be large or small.

The reason that even Bayesians turn to *simple* significance tests, if they want to check their assumptions, is that such tests do not require specifying an alternative, but only a single, appropriately chosen, null hypothesis (e.g., Bayarri & Berger, 2004; Box, 1983; Gelman, 2011; Gelman & Shalizi, 2013). Bayesian statistician George Box famously advocated eclecticism because “diagnostic checks and tests of fit which, I will argue, require frequentist theory significance tests for their formal justification” (Box, 1983, p. 57).

In testing assumptions, typically the null hypothesis is that the assumption(s) hold. Suppose in our example of testing the mean of a Normal distribution we want to test the assumption that the random variables are independent and identically distributed (IID), based on data set ${{\varvec{x}}}_{0}=\left({x}_{1},{x}_{2},...,{x}_{n}\right)$. We transform ***x***_0_ into “runs” by recording whether the difference between successive observations is positive ($+$) or negative ($-$). So for example if *x*_2_ is greater (less) than *x*_1_, the first element of our new data set is + ($-$), and so on for each *x*_i_. The data with 10 numbers might look like $+,+,-, -, +,+,+, +, -,-$. Each sequence of pluses only or minuses only is a *run*, so our new data shows 4 runs. The distribution of R, the number of runs, can be computed under the assumption that ***x***_0_ is a realization of an IID sample. It depends only on sample size n. The expected number of runs, under IID, is (2n − 1)/3. The standard error SE equals √(16n − 29)/90.^9^ IID is rejected if there are either too many or too few runs. This nonparametric test does not depend on the underlying distribution assumption. This allows falsifying assumptions, as well as pinpointing improved models. If an assumption passes a variety of probes, one can argue there is evidence that any departure from the assumption is not too dramatic, particularly with tests known to be robust to small departures from assumptions.

The intimate link between experimental design and interpretation in statistical significance testing is especially seen in design-based, as opposed to model based, p-values: “The simple precaution of randomisation will suffice to guarantee the validity of the test of significance, by which the result of the experiment is to be judged” (Fisher, 1935a, p. 24). Suppose a Covid treatment, say dexamethasone, makes no difference to hospitalized patients (receiving oxygen) (Horby et al., 2021). Then patients would have died or not within 28 days regardless of whether they were assigned to the treatment or control group. Under the sharp null hypothesis (of no effect), therefore, any observed differences between the two groups would be due to the accidental assignment to the treated or control groups. Thanks to the random assignment to the two groups we can determine the probability of any observed difference due to the accidental assignment of groups. This allows computing the statistical significance level, which then controls the Type 1 error probability.

## Criticisms and/or reforms that presuppose rival statistical philosophies

We have considered familiar criticisms of p-values based on misuses and have proposed a reinterpretation that readily avoids them. Other criticisms revolve around controversial presuppositions about the very concept of evidence and the appropriate roles of probability in statistical inference. These are associated with two main alternatives to the error statistical philosophy, likelihoodist and Bayesian accounts, although they come in many forms. Here probability is used to assign a degree of probability, confirmation, support or belief in hypotheses, given data ***x***_0_. The measure can be absolute, as in computing a posterior probability of a hypothesis, or comparative as with likelihood ratios, Bayes factors, and model selection. To have a single heading for these alternatives, we may place them all under the umbrella of *probabilisms* (although nothing turns on combining them). Even where today’s critics view themselves as merely objecting to misuses of tests, the proposed fixes and alternatives grow out of these philosophical presuppositions. Pointing to core assumptions is a way to quickly get to the heart of what may appear to be disconnected criticisms.

### P-values violate the likelihood principle

As Sober (2008, p. 77) observes, Bayesianism and likelihoodism “both interpret experimental results by using the law of likelihood”. The *law of likelihood* states: data ***x*** support *H*_1_ over *H*_0_ if the likelihood of *H*_1_ exceeds *H*_0_, that is, if the likelihood ratio Pr(***x***|*H*_1_)/Pr(***x***|*H*_0_) exceeds 1. The likelihood function is the probability (or density) of the *observed* value of the test statistic, regarded as a function of the unknown parameter(s). The *likelihood principle (LP),* which goes a bit further, asserts that all the evidence about the unknown parameter(s) resides in the likelihood ratio, once the data are observed.^10^ Based on assuming the LP, critics Burnham and Anderson charge that “P-values are not proper evidence as they violate the LP (Royall, 1997). Another way to understand this is the ‘irrelevance of the sample space principle’” (2014, p. 627).

The LP is violated by statistical significance testing. As with other error statistical methods, statistical significance tests are not based *solely* on the probability of the observed data but also consider the probability that one might have observed other values. The LP, by contrast, conditions on the observed data. *Likelihood analysis is answering a different question from statistical significance testing*, so that if one wishes to answer the statistical significance question, it will not do to follow the LP. Methods that obey the LP do not provide error control in the sense of the error statistician.^11^ A central problem is that any hypothesis that perfectly fits the data is maximally likely.^12^ One can therefore readily find in one’s data a better supported alternative than *H*_0_ since “there *always* is such a rival hypothesis viz*.*, that things just had to turn out the way they actually did” (Barnard, 1972, p. 129). In other words, there is a high probability that Pr(*H*_0_ is less well supported than *H*_1_;*H*_0_) for *some H*_1_ or *other*. That is why N–P say, in order “to fix a limit between ‘small’ and ‘large’ values of [the likelihood ratio] *we must know how often such values appear when we deal with a true hypothesis*” (Pearson & Neyman, 1930, p. 106).^13^

Leading likelihoodist Richard Royall himself gives the example of a “trick deck.” Having shuffled a deck of ordinary-looking playing cards; you turn over the top card and find an ace of diamonds: “According to the law of likelihood, the hypothesis that the deck consists of 52 aces of diamonds (*H*_1_) is better supported than the hypothesis that the deck is normal (*H*_N_) [by the factor 52]” (Royall, 1997, pp. 13–14). Although in such a case, an appeal to a prior disbelief scotches any inference to a trick deck, it is important to see that according to this account of evidence, the trick deck is still maximally supported.

Whatever one’s view, a criticism that presupposes the irrelevance of error probabilities to evidence is radically different from one that points to misuses of tests for their intended purpose—to assess and control error probabilities. Any “fix” based on satisfying the LP will not do the job of statistical significance tests.

### Statistical significance tests are not giving a comparative appraisal

We agree with Sober (2008, p. 77) that Bayesian and likelihoodist accounts should not be saddled “with ideas that are alien to them”, but think the same principle should apply in evaluating statistical significance tests. According to Sober, the fact that “significance tests don’t contrast the null hypothesis with alternatives suffices to show that they do not provide a good rule for rejection” (ibid., p. 56). But statistical significance testing is not of the comparative (“evidence favoring”) variety. To the significance tester this is a central asset rather than a liability. To merely infer that one hypothesis is more likely than the other by a given amount is not to provide evidence for or against either. Both can be unlikely.

Moreover, the comparative likelihoodist appraisal precludes the testing of *composite* hypotheses that are central to statistical significance testing—as in our earlier example where *H*_0_ asserts the vaccine efficacy δ ≤ 30% while *H*_1_ asserts δ > 30%. (A *simple* or *point* hypothesis, by contrast, would have an equality such as δ = 30%.) The fact that statistical significance tests generally test composite hypotheses becomes problematic for the strict likelihoodist. The problem, as the likelihoodist sees it, is that even though the likelihood of δ = 30% is small, there are values within alternative *H*_1_: δ > 30% that are even less likely on the data ***x*** that has reached a specified p-value. To get the point without computations, imagine an alternative hypothesizing 100% vaccine effectiveness. Such an extreme alternative would typically be *less* likely than the null hypothesis *H*_0_ that δ ≤ 30%. Should that preclude inferring *H*_1_? For the likelihoodist, rejecting *H*_0_: δ ≤ 30% and inferring *H*_1_: δ > 30% is to assert every parameter point within *H*_1_ is more likely than every point in *H*_0_. To the statistical significance tester, this seems an idiosyncratic meaning to attach to “infer evidence of δ > 30%”, but it explains a key objection raised by the likelihoodist. The significance tester just takes rejecting *H*_0_: δ ≤ 30% as inferring evidence of *some* positive discrepancy from 30%.

### The p-value is not the probability that a test (or null) hypothesis is true

A p-value of 0.05 means Pr(*d*(***X***) ≥ *d*(***x***_0_); *H*_0_) = 0.05. It is not the conditional probability Pr(*H*_0_|*d*(***X***) ≥ *d*(***x***_0_)). The latter is Pr(*d*(***X***) ≥ *d*(***x***_0_) and *H*_0_)/ Pr(*H*_0_) which requires a prior probability assignment to statistical hypothesis *H*_0_ and its alternatives. However, some critics charge that unless the p-value is mistakenly interpreted as a posterior probability, it is of questionable relevance to inference. That assumes a philosophy of inference at odds with statistical significance testing.

#### To a Bayesian, parameters are random

Jay Kadane, a subjective Bayesian, describes “[t]he key distinction between Bayesian and sampling theory statistics” [i.e., error statistics] as concerning the fundamental issue “of what is to be regarded as random and what is to be regarded as fixed. To a Bayesian, parameters are random and data, once observed, are fixed” (Kadane, 2011, p. 437). By contrast, Kadane says,“[t]o a sampling theorist, data are random even after being observed, but parameters are fixed” (ibid.). In particular, the probability statement Pr(*d*(***X***) > 1.96) = 0.025 “is a statement about *d*(***X***) before it is observed. After it is observed, the event [*d*(***X***) > 1.96] either happened or did not happen and hence has probability either one or zero” (ibid., p. 439).^14^ But this is incorrect: To sampling theorists, or error statisticians, the data which have been observed are also fixed, but they are still interested in the probability the test method would have resulted in an even greater observed *d*(***x***) even if *H*_0_ is true. They are interested in how *probative* their test was. Given how fundamentally different the style of reasoning, the Bayesian and error statistician are often talking past each other. The Bayesian is assuming *probabilism,* while the error statistician is assessing a method’s *probativeness*.

An example of how a criticism of statistical significance tests can grow out of assuming a perspective at odds with the fundamental philosophy underlying such tests, is the charge that p*-values exaggerate the evidence* against a null hypothesis (Berger & Sellke, 1987; Edwards et al., 1963). This boils down to the fact that a p-value may be low, pointing to evidence against *H*_0_, while a posterior probability on *H*_0_ might be high. The Bayesian criticism here makes the implicit assumption that posterior probabilities are the correct measure of evidence. Yet one could equally say that these posterior probability measures *understate* the evidence. The fact is that the different measures do different things.

#### Replace statistical significance

The recent popular movement to “Redefine Statistical Significance” (Benjamin et al., 2018), which garnered roughly 80 co-authors or signees, recommends that the conventional threshold of 0.05 be replaced with 0.005 so that it will better correspond to a high posterior probability on the alternative. To be clear, our objection is not to lowering the p-value threshold. We think thresholds are to be set in accordance with needs of given contexts. The problem is basing this reform on the supposition that a p-value should match Bayesian measures. Rather than call this a redefinition of statistical significance—since a particular threshold is not part of defining the concept—we refer to it as a call to *replace* a conventional threshold for statistical significance (using a Bayes factor or other probabilist measure as a reference point). A group response to Benjamin et al. (2018) is Lakens et al. (2018).

The result on which this call is based is actually quite old (Edwards et al., 1963). It turns on the Jeffreys–Lindley “paradox”, or more appropriately, the Fisher–Bayes disagreement between posterior probabilities and p-values. The classic example refers to testing the mean μ of a Normal distribution (with a known variance σ^2^). There is a point null hypothesis *H*_1_: μ = 0 and the alternative is *H*_1_: μ ≠ 0. A “lump” or “spike” of prior probability, say 0.5, is given to *H*_0_ (or a tiny region around 0), the remaining 0.5 is spread over the entire alternative space. As the sample size increases, even a result that is statistically significantly different from 0 can be more probable under *H*_0_ than under *H*_1_. However, it is important to see that p-values can also equal the posterior (with a diffuse rather than a spiked prior)—even though they measure very different things. These are sometimes called *frequentist matching* priors. Berger (2006), despite being one of the originators of this criticism of p-values, allows that the p-value is a good measure of evidence when one does not have a strong prior belief in the null hypothesis. (See also Sect. 5.3.)

Casella and R. Berger claim that “[c]oncentrating mass on the point null hypothesis is biasing the prior in favor of *H*_0_ as much as possible” (1987a, p. 111). They point out that the most common uses of a point null, asserting the difference between means is 0, merely describe a potentially interesting feature of the population, with “no a priori concentration of belief about *H*_0_” (Casella & Berger, 1987b, p. 345). They argue, “the very argument [that some critics] use to dismiss P-values can be turned around to argue for P-values” (ibid., p. 346). That is because in one-sided testing, without the spiked prior, the p-value can be “reconciled” with the posterior probability on the null hypothesis.^15^ As important as is this retort by Casella and R. Berger, the deeper problem is getting lost: the statistical significance test does not use prior probabilities, and is not seeking to assign a posterior probability to *H*_0_, (whether subjective, non-subjective, empirical or other). Stephen Senn gives a good upshot:

… [S]ome Bayesians in criticizing P-values seem to think that it is appropriate to use a threshold for significance of 0.95 of the probability of the alternative hypothesis being true. This makes no more sense than, in moving from a minimum height standard (say) for recruiting police officers to a minimum weight standard, declaring that since it was previously 6 foot it must now be 6 stone. (Senn, 2001, p. 202)

We grant that by setting the threshold to 0.005, along with certain choices of prior probabilities, the p-value can be made to correspond more closely to a posterior of 0.95 on a chosen alternative.^16^ But we deny this shows the p-value exaggerates evidence.^17^

Again, whether tests should use a lower Type 1 error probability is a separate issue. The problem is supposing there should be agreement between quantities measuring different things. Interestingly, an eclectic group of authors (in a supporting document for the 2016 ASA Statement), Greenland et al. (2016), concede, whether p-values exaggerate.

depends on one’s philosophy of statistics. …[M]any other statisticians do not accept these [Bayesian] quantities as gold standards, and instead point out that P values summarize crucial evidence needed to gauge the error rates of decisions based on statistical tests (p. 5).

See also Greenland (2019) and Haig (2020).

The claims in Sects. 4.1–4.3 are true, but they do not constitute criticisms of statistical significance tests from the perspective of the statistical significance tester. Any proposed reform that assumes these are bugs rather than features goes beyond pointing to misuses of p-values to importantly different conceptions of evidence and inference. Some maintain that problems with p-values are not about underlying philosophy, the problem is that they exaggerate evidence, or conflict with the LP, or with Bayesian posterior probabilities. But these are problems only under conceptions at odds with the one underlying statistical significance tests—call them what you wish.

Sections 3 and 4 do not cover all criticisms or proposed fixes. We will take up further criticisms in Sect. 5.

## Abandoning statistical significance tests is damaging scientific practice

In arguing our position, we must take those who advocate replacing, redefining or abandoning statistical significance at their word. We need to look at their actual arguments and recommendations and not dismiss the consequences of their platform because they only intend to stop abuses and misinterpretations. To change their arguments and recommendations to ones that would not be causing damage, would be to close one’s eyes to the damage that we argue is occurring.

### Researcher flexibility and data dredging are exacerbated

By and large, there is agreement as to the source of lack of replication. In many fields, latitude in collecting and interpreting data makes it too easy to dredge up impressive looking findings even when spurious. Any reasonably large data set will have interesting patterns or unusual data configurations which are merely the consequence of random variation (Hand, 2014). Such patterns, and their associated low p-values, will disappear when an independent group seeks to replicate the results—and the random variation goes in a different way. Or, to put it another way, the reported p-values are incorrect—they are not genuine p-values. The problem is not testing several claims, the problem is selectively reporting results: that is what causes high error probabilities.

Interestingly, even agreement on this source of poor replication has led to disagreement about dropping the use of p-value thresholds in interpreting results. A key argument made for dropping them is that without a p-value threshold the researcher would lose the (perverse) incentive to data dredge, p-hack, or try and try again when confronted with a statistically insignificant p-value. But it is not p-value thresholds which are the problem here: any measure for showing apparent structures in data is susceptible to the generation of spurious results via data dredging, and would be susceptible to the same perverse incentive. Insofar as p-values are still used (and nearly all proposals to replace statistical significance suggest retaining p-values), researchers would still need to show a reasonably small p-value to claim to have evidence of a genuine effect. Were they to claim large p-values supplied such evidence, they would be forced into the nonsensical position of saying “Even though more extreme results than ours would frequently occur by chance variability alone, I maintain our data provide evidence they are not due to chance variability.”

Researchers will still be keen to show evidence of an effect, be it positive or negative. The tendency to spin and selectively report on results is a well-researched empirical fact. Even in fields like medicine where there are official channels to monitor outcome-switching between pre-specified and reported results, “outcome misreporting continues to be prevalent” (Goldacre et al., 2019, p.1). Any researchers incentivized to data dredge in order to arrive at an apparently small p-value would be that much more incentivized to dredge were they assured they did not need to meet predesignated statistical significance level thresholds. (Granted they might not have to dredge as far.) That is because it would be hard to hold them accountable when they report data-dredged results in just the same way as if they had been predesignated. After all, what distinguishes nominal p-values from actual ones is that they fail to meet a prespecified error probability. Moreover, regarding p-values as “reasonably small” without explicitly stating a threshold is not equivalent to not using one, but rather means that the researcher has the freedom to bend their definition of “small” to achieve their aims. It means that any decision or recommendation arrived at is based on unclear, woolly, and unstated decision criteria. In an editorial in *Clinical Trials*, Cook et al., (2019, p. 224) worry that, “By removing the prespecified significance level, typically 5%, interpretation could become completely arbitrary. It will also not stop data-dredging, selective reporting, or the numerous other ways in which data analytic strategies can result in grossly misleading conclusions.”

#### The case of Harkonen

Even the earlier 2016 ASA Statement has been used as grounds to free researchers from culpability for failing to report or adjust for data dredging and multiple testing. In one case that reached the Supreme Court of the United States in 2009, Scott Harkonen (CEO of a drug company InterMune) was found guilty of wire fraud for issuing a misleading press report purporting that his company’s drug showed a survival benefit for a fatal lung disease. Downplaying the high p-value on the primary endpoint, that the drug improves lung function (and 10 secondary endpoints), he reported statistically significant drug benefits had been shown, without mentioning this referred only to a subgroup he identified from ransacking the unblinded data. Nevertheless, in the last of many years of appeals, Harkonen and his defenders argued that “the conclusions from the ASA Principles are the opposite of the government's" conclusion that his construal of the data was misleading (Harkonen v. United States, 2018, p. 16). The theory on which the client’s guilt rests—statistical significance tests—is declared to have been “shown false” by the 2016 ASA Statement. (For details see Mayo, 2020.)

It might be claimed that Harkonen’s defenders were deliberately distorting the 2016 ASA Statement. We think they are in earnest, and unfortunately the Statement provides them some grounds. Immediately after giving the 6 principles, the Statement notes “In view of the prevalent misuses of and misconceptions concerning p-values, some statisticians prefer to supplement or even replace p-values with other approaches”. Moreover, some of the “other approaches” listed do not uphold the statistical significance tester’s rules against multiple testing. It seems to us that “If the 2016 ASA guide opens the door to giving data dredging a free pass, the recommendation in [WSL 2019] swings the door wide open” (Mayo, 2020).

It is important to recognize that the problem of selective reporting and data dredging can occur when using Bayes factors, likelihood ratios, and other alternative methods. The inference will still be adversely affected even where the method lacks antennae to detect the problem, as with accounts that adhere to the LP (Sect. 4.1). Proponents of alternative methods may say, as they increasingly do, that they too will not allow ignoring multiplicity and selection effects, but that is not the same as having a rationale for taking them into account. (See Lakens, 2019.) If explicit principles are now to be added to those accounts, that is all to the good. That is where the focus of discussion on the reforms should lie.^18^

Familiar techniques to control overall Type 1 error rates rely on adjusting α thresholds for individual tests. Leading supporters of abandoning thresholds, Hurlbert et al. (2019) admit:

Others may be concerned about how we can justify and determine or fix set-wise or family-wise Type I error rates when multiple tests or comparisons are being conducted if we abandon critical *p*-values and fixed α's for individual tests. The short and happy answer is 'you can't. And shouldn't try!’ (Hurlbert et al., 2019, p. 354)

This is not reassuring for those who care about error probability control.

Commonly, challenges to adjusting for multiplicity come from those whose philosophical conception of evidence comports with the LP. Consider a remark by statistician Steven Goodman, a contributor to the ASA p-value project:

Two problems that plague frequentist inference: multiple comparisons and multiple looks, or, as they are more commonly called, *data dredging* and peeking at the data. The frequentist solution to both problems involves adjusting the *P* value…But adjusting the measure of evidence because of considerations that have nothing to do with the data defies scientific sense. (Goodman, 1999, p. 1010)

But does it defy scientific sense? To the error statistical tester, selection effects alter the error probing capacities of methods, and thus have everything to do with the data. Rather than see it as adjusting the p-value, it is more aptly seen as a matter of correctly computing the p-value. To be charitable, we may assume that Goodman also wants to take selection effects into account, but in some other way, perhaps via altered prior probabilities. But this needs to be shown. We might then compare which method gives the more direct way of taking into account the information about selection effects.

#### Bayesian clinical trials

Stopping at interim points of clinical trials is common for both frequentists and Bayesians, but to control the Type 1 error probability it is required to prespecify stopping points and adjust p-values according to the stopping rule. This puts advocates of Bayesian clinical trials in a quandary because,

the [regulatory] requirement of type I error control for Bayesian [trials] causes them to lose many of their philosophical advantages, such as compliance with the likelihood principle, and creates a design that is inherently frequentist … That is, inferential corrections, e.g., adjustments to posterior probabilities, are not required for multiple looks at the data. (Ryan et al., 2020, p. 7)

They admit that the “type I error was inflated in the Bayesian adaptive designs through incorporation of interim analyses that allowed early stopping for efficacy and without adjustments to account for multiplicity.” (They separately consider stopping for futility.) Yet they suggest that: “Given the recent discussions to abandon significance testing it may be useful to move away from controlling type I error entirely in trial designs” (ibid., p. 7). The high error probability would still be there, but would not be reported in the posterior probability. To some this is welcome; to others, it seriously damages error control.

### A central function of statistics in science is undermined

WSL 2019 do not restrict their recommendations to particular fields, say to observational studies lacking controls offered by randomized controlled trials (RCTs). They call for abandoning statistical significance across all science, acknowledging that strict compliance with the no threshold view is incompatible with the FDA practice of using predesignated thresholds in Phase III trials. (Generally a drug must show statistical significance at a small value in two trials before it will be accepted by the FDA.)

In 2019, *The New England Journal of Medicine* (*NEJM*) resists WSL’s 2019 call to abandon statistical significance thresholds, asserting:

A well-designed randomized or observational study will have a primary hypothesis and a prespecified method of analysis, and the significance level from that analysis is a reliable indicator of the extent to which the observed data contradict a null hypothesis of no association between an intervention or an exposure and a response. Clinicians and regulatory agencies must make decisions about which treatment to use or to allow to be marketed, and P values interpreted by reliably calculated thresholds subjected to appropriate adjustments [for multiple trials] have a role in those decisions. (Harrington et al., 2019, p. 286)

The WSL 2019 position does not merely object to having a single threshold, but to any number of thresholds. “[T]he problem is not that of having only two labels. Results should not be trichotomized, or indeed categorized into any number of groups…” (p. 2). This glosses over the fact that all real data are necessarily grouped—they are measured to a finite number of decimal places. Note too that the use of classification thresholds is not in opposition to also reporting the particular measure reached. We employ thresholds to distinguish myriad characteristics: high and low blood pressure, PSA levels in prostate cancer, etc.—even though a person’s actual reading is also reported. One might say the cut-offs are conventions, but this does not make them arbitrary.

Even the use of confidence intervals, advocated by many as a replacement for statistical significance tests, would violate the rule that results should not be “categorized into any number of groups”. An objection to taking a difference that reaches p-value 0.025 as evidence of a discrepancy from the null hypothesis would also be an objection to taking it as evidence that the parameter exceeds the lower 0.025 CI bound (or is “incompatible,” at that level, with the parameter values below it). They are identical, insofar as CIs retain their duality with statistical tests. Do any statistical tests survive the no threshold rule? It seems to us that without thresholds there are no tests.

The possibility of falsification is what distinguishes good science from questionable science. While falsification need not be a binary decision (one can have an intermediate zone requiring more evidence to be gathered), there needs to be some point for distinguishing data which are seriously inconsistent with a test hypothesis *H* from those that corroborate it. What is the point of insisting on replication checks if researchers can always deny their effects have failed to replicate? We agree with John Ioannidis that “fields that obstinately resist refutation can hide behind the abolition of statistical significance but risk becoming self-ostracized from the remit of science” (2019, p. 2068). However, if influential voices reject p-value thresholds, there is a danger that the grounds for ostracism might evaporate.

So that there is no misunderstanding, there are contexts where the goal is constructing a theory to be tested on other data, sometimes called an *exploratory inquiry*. (See Hand, 1994.) The reason new data are needed to check hypotheses gleaned from exploration is precisely that finding a hypothesis that fits the data is different from testing it.

It might be said that even those espousing the no threshold view do not *really* favor an end to the use of thresholds in inferences (outside of contexts of inductive behavior). If they do not, they should not be saying it. Their recommendations are being taken to heart every day in science, business, law and elsewhere. Although statistical falsification does not mean that further evidence might not reverse that decision, for a given test of *H* it is essential to specify in advance at least some outcomes that will not be allowed to count in favor of *H*. That is to have a threshold.

### Recommendations to replace, abandon or retire statistical significance do not do the job of statistical significance tests

The ASA Executive Director's editorial (WSL 2019) admits the statistical community does not agree about statistical methods, and “in fact it may never do so” (p. 2). To nevertheless call for ousting statistical significance tests, or their thresholds, from the large variety of tools that practitioners regularly use is unwarranted and is damaging. (See Mayo, 2022.) Let us examine some of the leading recommendations to retire, replace/redefine, and abandon statistical significance to cash out our charges in Sects. 5.1 and 5.2 (that they exacerbate researcher flexibility, and undermine a key function of statistics in science).

#### Retire

Begin with the recommendation that is closest to the error statistical paradigm: Amrhein et al.’s (2019) “retire statistical significance*”,* the article enlisted by *Nature* to amplify WSL 2019. For convenience, we refer to it as (AGM), the initials of its authors Amrhein, Greenland, McShane. They recommend reporting the 0.95 confidence interval, although they rename them compatibility intervals—redolent of the "consonance intervals" of Kempthorne and Folks (1971). The latter has the advantage of using many thresholds, one for each of several consonance levels, while AGM use the classic 0.95 level. Where the N–P tester infers the data are statistically significantly different (usually indicating the direction) from a hypothesized reference point (at the given level, e.g., 0.05), AGM may report that the data are not compatible with the parameter values outside the 0.95 interval. Insofar as they choose a threshold, they could do the job of statistical significance tests. But a mere comparative report of compatibility (e.g., those within the interval are more compatible than those outside) would not deem any hypotheses about parameters incompatible with the data (however high the confidence level). We think there is a role for first identifying an effect as genuine (via a significance test) before estimating its effect size. Additional reasons we find AGM’s “retire” to be damaging are as follows.

First, echoing WSL 2019, AGM declare: “Decisions to interpret or to publish results will not be based on statistical thresholds”. As argued in Sect. 5.1, this gives a pretext to the data-dredger to wriggle out of charges that they have failed to meet a predesignated threshold. They can conveniently declare: “Decisions to interpret results are not supposed to be based on thresholds, so how can I be blamed for not upholding one?”

Second, they recommend that p-values be restricted to behavioristic, performance goals. Say AGM: “We are not calling for a ban on *P* values. Nor are we saying they cannot be used as a decision criterion in certain specialized applications (such as determining whether a manufacturing process meets some quality-control standard)” (p. 306). However, scientists use p-values for the general goal of finding things out: Do the data indicate that dexamethasone has a statistically significant survival benefit for hospitalized patients (receiving oxygen)? Are these data evidence of a Higgs particle? To maintain that p-values (or significance tests) be restricted to routine quality control contexts would rob scientists of a general tool for dealing with random error. Of course scientists typically must show the statistically significant effect in several trials before claiming evidence of a genuine effect—but that does not turn them into quality control tools, where only performance matters.

Third, what justifies the CI compatibility report that the parameter, say μ, is within the particular CI formed? Given how much importance critics place on misinterpreting p-values, one would have expected this question to be addressed in proposed reforms. An extremely common fallacy is to suppose that the 0.95 assigns a probability to the particular CI formed. For example, it might be claimed that the probability is 0.95 that μ is in the interval, say, [0, 4]. This is incorrect. At most AGM can say that this particular interval arose from a procedure that in the long run would cover the true parameter value with probability 0.95. This is a quality control performance assessment that they purport to want scientists to move away from! The statistical significance tester, by contrast can give an inferential rationale of this form: The reason the data are evidence μ > 0 is that, were μ ≤ 0, then with high probability we would have gotten a smaller ***x*** than we did. This probability is still operative, post-data, and is the key to the severity interpretation of tests.

Perhaps they regard their CIs as merely descriptive, without enjoying an associated 0.05 error probability. WSL 2019 may already be having the effect, likely unintended, of divorcing CIs from their initial error probability guarantees. The NEJM’s revised guidelines (2019) stipulate: When no method to adjust for multiplicity of inferences or controlling the Type 1 error probability is prespecified, the report of secondary endpoints:

should be limited to point estimates of treatment effects with 95% confidence intervals. In such cases, the Methods section should note that the widths of the intervals have not been adjusted for multiplicity and that the inferences drawn may not be reproducible. No P values should be reported for these analyses.

Severing CIs from their dualities with tests, we think, is damaging.

#### Replace/redefine

Move now to assessing Bayesian replacements. This requires a practitioner to know how to interpret the prior probabilities involved. Some might take priors to measure strength of belief, which a tester might feel are quite fragile. One can hold strong beliefs in a hypothesis which has been subjected to weak tests. A more prevalent view appeals to non-subjective or default priors. They are intended to let the data be dominant in some sense. “Technically they are only positive functions to be formally used in Bayes’ theorem to obtain ‘non-subjective posteriors’...” (Bernardo, 1997, pp. 159–60). The question of interpretation looms large. “If the prior is only a formal device and not to be interpreted as a probability, what interpretation is justified for the posterior as an adequate summary of information?” (Cox, 2006, p. 77). (The error statistician will use frequentist priors when they are available and testable.)

We have discussed aspects of the “replace/redefine” significance in Sect. 4.3. Our focus here is on the particular recommendation put forward within the recent, highly influential call to “redefine” statistical significance (Benjamin et al., 2018; Ioannidis, 2005; Johnson, 2013). It has its roots in a much broader Bayes factor approach pioneered by Berger (2003, 2006). It has found its way into such popular treatises as the 2019 National Academy of Science (NAS) Consensus Study Report on Replication. The idea is to replace p-values with the kind of assessment from diagnostic screening in medicine. Call it the *diagnostic screening* replacement. The outcomes are dichotomized into two: statistically significant (e.g., at the 0.005 level) or not, the significance level having been fixed. In this replacement, assessing evidence for *H*_1_ is to be obtained by the posterior prevalence of *H*_1_, or the *posterior predictive value* (PPV).^19^ In other words, a threshold remains, e.g., 0.005, but its occurrence becomes an event in a Bayesian (or quasi-Bayesian) computation of a posterior probability. (See Mayo, 2018.)

Suppose our practitioner is facing a result statistically significant at the 0.05 level, which means it reaches the 1-sided level of 0.025. According to Benjamin et al. (2018), the practitioner should report that there is only weak evidence against the null hypothesis, unless one has a high prior degree of belief that the effect is present—that is, a low probability on *H*_0_ of “no effect”. Otherwise, a null hypothesis *H*_0_ of “no effect” is given a high prior probability (e.g., 0.8, 0.9). This prior probability may arise in an unusual, quasi-frequentist manner. (There are generally just two hypothesis: *H*_0_ and *H*_1_.) It is assumed *H*_0_ was selected (randomly?) from a population of null hypotheses with a high prevalence (e.g., 90%) of truth. This renders *H*_0_ probable, *H*_1_ improbable. Not only would this prior prevalence be unknown, the attempt is vitiated by the *reference class* problem. Should we look at the prevalence of “no effects” within a given research area? Within a given type of study, e.g., observational, randomized control? Moreover, from the fact that *H* comes from a pool where k% are true, we do not get the probability that this particular *H* is true. Such an assignment is fallacious, for the same reason a confidence level is not the probability a particular interval is true. Computing a PPV is apt in given contexts of predicting the prevalence of properties, e.g., the presence of disease in high throughput screening, but it does not provide an assessment of plausibility or well-testedness of a particular hypothesis.

Ioannidis (2005) proposed assigning priors according to the group a hypothesis is thought to fall into: high priors for those examined via RCTs, low priors for exploratory research and discovery. Assigning prior probabilities by dint of “association” might have the damaging consequence of disincentivizing both groups to avoid bias: for the former (latter), because assigning priors by group means they will be accorded fairly high (low) prior prevalence regardless of the effort made in the case at hand. (Further criticisms of the diagnostic screening replacement are Goodman & Greenland, 2007; Mayo, 2018; Spanos, 2010).

Granted, if the concepts of N–P power and the Type 1 error probability are allowed to be treated as likelihoods in a Bayesian computation, lowering the p-value threshold (e.g., to 0.005) gets a justification in terms of raising the PPV (see footnote 19). This can have the odd effect of giving a high PPV to alternatives tested with high power. There is no problem if power is assumed to be the same for all alternatives (as Benjamin et al., 2019 assume), but it entirely changes the assessment of the warrant to accord the particular effect and research effort under study. Ironically, it becomes more like the quality control assessment that statistical significance test critics deride. Finally, its advocates admit that:

The proposal does not address multiple hypothesis testing, P-hacking, publication bias, low power, or other biases … which are arguably the bigger problems. We agree. Reducing the P value threshold complements — but does not substitute for — solutions to these other problems. (Benjamin et al., 2018, p. 8)

So a practitioner embracing “redefine/replace” still needs to appeal to methods to address multiplicity and the other “bigger problems”. But these bigger problems are the ones underlying the replication crisis, and the error statistical account supplies ways to address them—and without prior probability assignments.^20^

#### Abandon

Suppose now our practitioner turns to a recommendation to “abandon” statistical significance in McShane et al. (2019)—one of the leading papers in the collection introduced by WSL 2019. According to McShane et al. (2019), problems are “unresolved by proposals involving modified p-value thresholds, confidence intervals, and Bayes factors” (p. 235). So the practitioner is not advised to use any of these. They are especially hard on the replace/redefine approach just discussed (Benjamin et al., 2018), because it retains p-value thresholds. Moreover they criticize it because “it falls short of being truly Bayesian” (p. 242). For example “it does not condition upon the actual observations but instead integrates over the observation space and hence may fall afoul of the likelihood principle” (ibid.). But, as we have already noted, any account that obeys the LP violates error statistics principles. Hearing them laud the LP, the practitioner is rightly worried that their recommendations will not control error probabilities.

McShane et al. (2019, p. 235) aver “that it seldom makes sense to calibrate evidence as a function of p-values or other purely statistical measures”. In our view, the p-value is an apt measure of evidence when engaged in the question of whether there is evidence of a genuine effect. The p-value is not “purely” formal: it requires knowledge of the context, how many tests were done, if there was data-dredging, and aspects of the design of the data generation (2016 ASA Statement, principle 4). The main positive proposal that McShane et al. (2019) offer the significance tester is to take account of a variety of background factors rather than looking only at the p-value. Of course that is right, and their list is clearly useful. But most practitioners will be aware that the error statistical framework contains systematic means for taking these factors into account in design and specification, and in multiplicity adjustments. So they will wonder what advantage this position holds, given we are not told how the abandoners propose to do so (except for an allusion to “informative priors”). The practitioner is at sea. She is left wondering how to proceed to answer her question of whether there is reasonably good evidence that a given intervention has a genuine positive effect. She recognizes there is much more to scientific inference than p-values—indeed, much more than statistics—but she seeks a way to ask questions piecemeal, rather than reach some grand substantive conclusion, all at once.

## Conclusion

A long paper calls for an overview. Allow us to supply one.

### Summary

As noted in Sect. 1, our paper explains why some of today’s attempts to fix statistical practice by abandoning or replacing statistical significance are actually jeopardizing reliability and integrity. The ASA Executive Director’s editorial, WSL 2019, is a vehicle to zero in on the current debates. In Sect. 2 we set out the main features of statistical significance tests, emphasizing aspects routinely misunderstood especially by their critics. Section 3 describes five mistaken interpretations of p-values around which numerous criticisms revolve, and shows how the improved formulation in Sect. 2 avoids them. These criticisms and especially proposed reforms are often intertwined with rival underlying conceptions about evidence and the role of probability in statistical inference. We call these the method’s “philosophy”. In Sect. 4, we delineate some of these rival philosophical conceptions. Even where critics of statistical significance tests are mainly objecting to misuses, recommended fixes often reflect underlying conceptions about evidence and probability. In Sect. 5 we employ the insights from the previous sections to argue that calls to abandon or replace statistical significance tests are damaging to scientific practice. We explain why banning the use of p-value thresholds does not diminish but rather exacerbates data-dredging and biasing selection effects (5.1), and undermines a central function of statistical testing in science (Sect. 5.2). Section 5.3 shows how the specific recommendations to retire, replace, or abandon statistical significance exemplify the problems in Sects. 5.1 and 5.2.

### A reviewer objection

One reviewer has objected that the damages we consider would only arise if the replace, retire, abandon statistical significance advocates enforced their view, precluding even thoughtful and appropriate uses of statistical significance testing. We disagree, and we have argued in detail why (see especially Sect. 5.3). Policies need not preclude valuable tools to result in undermining them. If legitimate characteristics, essential to the successful use of significance tests, are the focus of criticism and disparagement, then the goals of tests are diminished, insofar as criticisms are attended to—which we think they are.

For example, thoughtful tests turn on specifying ahead of time outcomes that will not be allowed to count in favor of a claim—but this is to identify a predesignated threshold, going against the “no threshold" view. Or again, essential goals of significance tests are weakened if tests are criticized for not obeying the LP, which finds error probabilities irrelevant, or if p-values are appraised using Bayesian quantities that are measuring different things. To recommend replacements, without acknowledging where such replacements lack essential features for accomplishing the tasks of significance tests, may discourage researchers from performing a key job of statistics in science.

#### Final remarks

P-values have the intrinsic properties for their task, if used properly. We have an indication of inconsistency with a test hypothesis *H*_0_ only when *H*_0_ very probably would have survived the test, if adequate, and yet the test yields results discordant with *H*_0_. The more probable it is that a test would have correctly alerted us that results are reasonably consistent with *H*_0_, the tougher the test that *H*_1_ *has* *passed*, and the stronger the evidence against *H*_0_. The same pattern of reasoning occurs at multiple stages of learning from data, formal or informal.

The debate is not over a single tool, it is much more than that. Statistical significance tests are a small part of a rich repertoire of methods, entwining design, modeling and inference, that have been developed to put deliberate constraints on human biases to construe data in terms of a preferred claim. It serves as a small piece contributing to full-bodied inquiries built on piecemeal error control. Many criticisms focus on those who take a single, isolated statistically significant result as evidence of a genuine experimental effect, and even of a substantive scientific theory. But the fact that a tool can be misunderstood and misused is not a sufficient justification for discarding that tool. Rather, methods for calling out and avoiding such mistakes are required—and error statistical principles enable just that.

The 2016 ASA Statement declared itself concerned that irreplication would lead to “doubt about the validity of science” (p. 129). To reject or play down the role of statistical significance tests risks implying that the statistical community accepts that those tools are unsuitable, rather than that misuse of those tools is the problem. The consequence could be “the most dramatic example of a scientific discipline shooting itself in the foot” (Hand, 2021). We know irreplications and fraud are unearthed by statistical significance tests, and that they are the basis for tests of assumptions of statistical models, which all accounts use. The replication crisis should not be used to replace statistical significance tests with alternative methods that do not accomplish statistical significance testing tasks.

Our critical analysis has implications for how to evaluate statistical methods with integrity. When WSL 2019 criticize “the seductive certainty falsely promised by statistical significance,” (p. 3), it is a shock to serious practitioners. Statistical tests provide certainty only in the hands of those who are misusing them. Indeed, it is the essence of statistical inference that it does *not* provide certainty. Perhaps they imagine the only way to express uncertainty is using comparative likelihoods, Bayes factors, or posterior probabilities (*probabilisms*). The error statistician instead qualifies the inference by assessing the error probing capabilities of the method used and the data that result (*probativism*). The WSL 2019 authors would have strengthened their cause had they given a more charitable interpretation of tests, avoiding straw person fallacies.

WSL 2019 tells us that “a declaration of ‘statistical significance’ has today become meaningless” (p. 2). Admittedly, a statistically significant p-value only gets its meaning in context, but it is not meaningless. Today, it is rather informative to learn, for example, that a given Covid-19 treatment yields a statistically significant decrease in mortality (at a specified level) between patients randomized to treatment versus control groups. WSL 2019 maintain “No *p*-value can reveal the …presence… of an association or effect” (p. 2), but this even conflicts with Principle 1 of the 2016 ASA Statement, that “*p*-values can indicate how incompatible the data are with a specified statistical model” (p. 131). An indication of how incompatible data are with a claim of the absence of a relationship *is* an indication of the *presence* of the relationship.

It is generally agreed that a large part of the blame for lack of replication in many fields may be traced to biases encouraged by the reward structure. The pressure to publish, to advance one’s career, is thought to seduce even researchers aware of the pitfalls of capitalizing on selection biases. That mindset makes for a highly susceptible group. It is risky to stand in opposition to journals and leaders of professional organizations. Instead of critically reflecting on the arguments independently, practitioners may blithely repeat the same criticisms, and accept that statistical significance tests have brought about a crisis in science. The ASA President’s Task Force is to be commended for distinguishing the Executive Director’s editorial (WSL 2019) from ASA policy. (See Mayo, 2022.) Rather than follow the most valuable recommendation of WSL 2019—that researchers take a neutral stance in confronting controversial hypotheses (part of their call for “Modesty”)—antagonists to statistical significance repeat the criticisms we have discussed, rarely engaging existing counterevidence in favor of statistical significance.^21^ Thus, the very process used today to advance a position purporting to improve on replication may inculcate the bad habits that lead to irreplication. This is another reason that calls to abandon statistical significance are damaging scientific practice.

## Footnotes

1 The six Principles from the 2016 ASA Statement on *p*-values (pp. 131–132):

1. *P*-values can indicate how incompatible the data are with a specified statistical model.
2. *P*-values do not measure the probability that the studied hypothesis is true, or the probability that the data were produced by random chance alone.
3. Scientific conclusions and business or policy decisions should not be based only on whether a *p*-value passes a specific threshold.
4. Proper inference requires full reporting and transparency.
5. A *p*-value, or statistical significance, does not measure the size of an effect or the importance of a result.
6. By itself, a *p*-value does not provide a good measure of evidence regarding a model or hypothesis.

2 They also should not be using “likely”. Although in ordinary English we use “probable” and “likely” interchangeably, in statistics they have very different meanings. The likelihood of a hypothesis given data ***x*** measures the probability or density of ***x*** computed under that hypothesis. Unlike with probability, several incompatible hypotheses can all have high or even maximal likelihood.

3 *H*_1_ is made up of all positive discrepancies from 30%, it is composite. Each particular value may be written as δ′.

4 This includes not only Bayesian accounts but Fisherian fiducial accounts. See Mayo (2018, Excursion 5).

5 The standard Normal variate Z is $(\overline{\text{X}}-\mu)/{\text{SE \, which \, would \, be\,}}(2-3)/1=-1.\;{\text{Pr}}({\text{Z}}>-1)=0.84.\;\;1{\text{SE}}=\upsigma/\surd n.$

6 An approach that gives confidence intervals for all confidence levels is that of confidence distributions, first suggested in Cox (1958) and developed more recently in Thornton and Xie (2022).

7 There is always a small enough discrepancy from a null such that the power to detect it is small—close to α.

8 This is discussed by Hanan Selvin in Morrison and Henkel’s classic volume, *The Significance Test Controversy* (1970). He also notes that if a sufficient number of the N data-dredged hypotheses reach nominal significance, the overall or family-wise *p*-value may still be low.

9 The test statistic for the test of IID is [R − E(R)]/SE. For a full discussion see Spanos (2019, Chaps. 5 and 15).

10 Likelihoods rest on assuming the statistical model.

11 Berger (2003) offers a Bayesian notion of “error probability” whereby the error probability associated with inferring *H* is its posterior probability. A discussion is in Mayo (2018, Excursion 3 Tour II).

12 In the case of two predesignated point hypotheses, *H*_0_: μ = μ_0_, and *H*_1_: μ = μ_1_, the error probabilities are controlled. In particular, the probability of obtaining a result that makes *H*_1_ r times more likely than *H*_0_, if *H*_0_ is true, is less than 1/r: Pr(LR > r; *H*_0_) < 1/r.

13 Ian Hacking based his logic of support on the law of likelihood (1965), but then rejected it in (1980) declaring “I now believe that Neyman, Peirce, and Braithwaite were on the right lines to follow in the analysis of inductive arguments” (p. 141). Still, Hacking did not commit to any one view.

14 The problem of how a Bayesian confirms *H* with data accorded a probability of 1, the *known evidence problem,* goes beyond the current discussion. (See Glymour, 1980.)

15 We agree with Cox and Hinkley (1974) that insofar as one is interested in the direction of the effect, and will not simply infer there is an effect in either direction, the two-sided test should be seen as combining two one-sided tests, doubling the p-value for a selection effect.

16 “A two-sided P value of 0.005 corresponds to Bayes factors between approximately 14 and 26 in favour of *H*_1_. This range represents ‘substantial’ to ‘strong’ evidence according to conventional Bayes factor classifications” (Benjamin et al., 2018). For computations and further references also see Edwards et al. (1963), Berger and Sellke (1987), Johnson (2013), Mayo (2018). See also the computation in footnote 19.

17 Bickel (2021) argues that “if the *p*-value is sufficiently small while the posterior probability according to a model is insufficiently small, then the model will fail a model check” and may need revision (p. 249). In this view, akin to Fisher (1956), conflicts between posteriors and p-values may be resolved by revising the Bayesian model.

18 If all hypotheses to be considered are already pre-specified in the model prior to the data, the Bayesian may claim to avoid a problem of selection effects in computing a posterior probability.

19

$$\mathrm{Pr}\left({H}_{1}|{\varvec{x}}\right)= \frac{\mathrm{Pr}\left({\varvec{x}}|{H}_{1}\right)\mathrm{Pr}({H}_{1})}{\mathrm{Pr}\left({\varvec{x}}|{H}_{1}\right)\mathrm{Pr}\left({H}_{1}\right)+\mathrm{Pr}\left({\varvec{x}}|{H}_{0}\right)\mathrm{Pr}({H}_{0})}$$

With Pr(*H*_1_) = 0.1, Pr(*H*_0_) = 0.9, and obtaining Pr(***x***|*H*_1_) = 0.9 and Pr(***x***|*H*_0_) = 0.05 from a test’s power and Type 1 error probability, respectively, the PPV is 0.64. Note that if Pr(***x***|*H*_0_) = 0.005, the Pr(*H*_1_|***x***) becomes 0.95.

20 We do not rule out ways to reconcile Bayesian and error statistical approaches using frequentist priors (Bayarri & Berger 2004; Efron, 2005) and assessing associated error probabilities.

21 While it is impossible to give a full list, we respond to a reviewer by giving a few: Birnbaum (1977), Cox (1977, 2006), Fisher (1935a, 1935b), Fraser (2011), Gelman and Shalizi (2013), Giere (1976), Haig (2016), Kempthorne and Folks (1971), Lehmann (1993, 2011), Mayo (1996, 2018), Mayo and Cox (2006), Neyman (1977), Senn (2002, 2007, 2011), Wellek (2017).

## References

[CR1] Altman D, Bland J (1995). Absence of evidence is not evidence of absence. BMJ.

[CR2] Amrhein V, Greenland S, McShane B (2019). Comment: Scientists rise up against statistical significance. Nature.

[CR3] Barnard G (1972). The logic of statistical inference (Review of “The Logic of Statistical Inference” by Ian Hacking). British Journal for the Philosophy of Science.

[CR4] Bayarri M, Berger J (2004). the interplay of Bayesian and frequentist analysis. Statistical Science.

[CR5] Benjamin D, Berger J, Johannesson M (2018). Redefine statistical significance. Nature Human Behaviour.

[CR6] Benjamini, Y. (2016). It’s not the *P*-values’ fault comment on *“Wasserstein, R. and Lazar, N. (2016), The ASA’s statement on p-values: Context, process and purpose. The American Statistician, 70(2), 129–133”. On-line supplemental material, 3rd item:*10.1080/00031305.2016.1154108?scroll=top*.*

[CR7] Benjamini Y, De Veaux R, Efron B (2021). The ASA President’s task force statement on statistical significance and replicability. The Annals of Applied Statistics.

[CR8] Benjamini Y, Hochberg Y (1995). Controlling the false discovery rate: A practical and powerful approach to multiple testing. Journal of the Royal Statistical Society B.

[CR10] Berger, J. (2003). Could Fisher, Jeffreys and Neyman have agreed on testing?’ and ‘Rejoinder’, *Statistical Science, 18*(1), 1–12, 28–32. 10.1214/ss/1056397485

[CR11] Berger, J. (2006). The case for objective Bayesian analysis and rejoinder. *Bayesian Analysis, 1*(3), 385–402, 457–464. 10.1214/06-BA115

[CR9] Berger, J., & Sellke, T. (1987). Testing a point null hypothesis: The irreconcilability of p values and evidence (with discussion and rejoinder). *Journal of the American Statistical Association, 82*(397), 112–122, 135–139. 10.2307/2289131

[CR12] Bernardo J (1997). Non-informative priors do not exist: A discussion. Journal of Statistical Planning and Inference.

[CR13] Bickel DR (2021). Null hypothesis significance testing defended and calibrated by Bayesian model checking. The American Statistician.

[CR14] Birnbaum A (1977). The Neyman–-Pearson theory as decision theory, and as inference theory; with a criticism of the Lindley–Savage Argument for Bayesian Theory. Synthese.

[CR15] Box, G. (1983). An apology for ecumenism in statistics. In G. Box, T. Leonard, & D. Wu (Eds.), *Scientific inference, data analysis, and robustness* (pp. 51–84.). Academic Press. 10.1016/B978-0-12-121160-8.50009-0

[CR16] Burnham, K., & Anderson, D. (2014). P values are only an index to evidence: 20th- vs. 21st-century statistical science. *Ecology, 95*(3), 627–630. 10.1890/13-1066.1 10.1890/13-1066.1 24804444

[CR17] Casella, G., & Berger, R. (1987b). Comment on testing precise hypotheses by J. O. Berger and M. Delampady. *Statistical Science, 2*(3), 344–347.

[CR18] Casella G, Berger R (1987). Reconciling Bayesian and frequentist evidence in the one-sided testing problem. Journal of the American Statistical Association.

[CR19] Cook J, Fergusson D, Ford I, Gonen M, Kimmelman J, Korn E, Begg C (2019). There is still a place for significance testing in clinical trials. Clinical Trials.

[CR20] Cox DR (1958). Some problems connected with statistical inference. The Annals of Mathematical Statistics.

[CR21] Cox DR (1977). The role of significance tests (with discussion). Scandinavian Journal of Statistics.

[CR22] Cox, D. R. (2006). *Principles of statistical inference*. Cambridge University Press. 10.1017/CBO9780511813559

[CR23] Cox, D. R., & Hinkley, D. (1974). *Theoretical statistics*. Chapman and Hall Ltd. 10.1201/b14832

[CR24] Edwards W, Lindman H, Savage L (1963). Bayesian statistical inference for psychological research. Psychological Review.

[CR25] Efron B (2005). Bayesians, frequentists, and scientists. Journal of the American Statistical Association.

[CR26] FDA (U. S. Food and Drug Administration). (2017). *Multiple endpoints in clinical trials: Guidance for industry (DRAFT GUIDANCE)*. Retrieved from https://www.fda.gov/media/102657/download

[CR27] Fisher, R. A. (1935a). *The design of experiments*. Oxford University Press.

[CR28] Fisher RA (1935). The logic of inductive inference. Journal of the Royal Statistical Society.

[CR29] Fisher, R. A. (1956). *Statistical methods and scientific inference*. Oliver and Boyd.

[CR30] Fraser, D. (2011). Is Bayes posterior just quick and dirty confidence? and rejoinder. *Statistical Science, 26*(3), 299–316, 329–331. 10.1214/11-STS352

[CR32] Gelman A (2011). Induction and deduction in Bayesian data analysis. Rationality, Markets and Morals (RMM).

[CR33] Gelman A, Loken E (2014). The statistical crisis in science. American Scientist.

[CR31] Gelman, A., & Shalizi, C. (2013). Philosophy and the practice of Bayesian statistics and Rejoinder. *British Journal of Mathematical and Statistical Psychology, 66*(1), 8–38, 76–80. 10.1111/j.2044-8317.2011.02037.x, 10.1111/j.2044-8317.2012.02066.x 10.1111/j.2044-8317.2011.02037.x PMC4476974 22364575

[CR34] Giere, R. (1976). Empirical probability, objective statistical methods, and scientific inquiry. In W. Harper & C. Hooker (Eds.), *Foundations of probability theory, statistical inference and statistical theories of science* (Vol. 2, pp. 63–101). D. Reidel. 10.1007/978-94-010-1436-6_3

[CR35] Glymour, C. (1980). *Theory and evidence*. Princeton University Press.

[CR36] Goldacre B (2019). COMPare: A prospective cohort study correcting and monitoring 58 misreported trials in real time. Trials.

[CR37] Goodman, S. (1999). Toward evidence-based medical statistics. 2: The Bayes factor. *Annals of Internal Medicine, 130*(12), 1005–1013. 10.7326/0003-4819-130-12-199906150-00019 10.7326/0003-4819-130-12-199906150-00019 10383350

[CR38] Goodman, S., & Greenland S. (2007). Assessing the unreliability of the medical literature: A response to “Why Most Published Research Findings Are False”. Johns Hopkins University, Department of Biostatistics Working Papers. Working Paper 135, pp. 1–25.

[CR40] Greenland S (2019). Valid p-values behave exactly as they should: Some misleading criticisms of p-values and their resolution with s-values. American Statistician.

[CR39] Greenland, S., Senn, S., Rothman, K., Carlin, J., Poole, C., Goodman, S., & Altman, D. (2016). Statistical tests, P values, confidence intervals, and power: a guide to misinterpretations comment on ‘Wasserstein, R. and Lazar, N. (2016), The ASA’s statement on p-values: Context, process and purpose. *The American Statistician, 70*(2), 129–133’. On-line supplemental material, 1st item. 10.1080/00031305.2016.1154108?scroll=top 10.1007/s10654-016-0149-3 PMC4877414 27209009

[CR42] Hacking, I. (1965). *Logic of statistical inference*. Cambridge University Press.

[CR41] Hacking, I. (1980). The theory of probable inference: Neyman, Peirce and Braithwaite. In D. Mellor (Ed.), *Science, belief and behavior: Essays in honour of R. B. Braithwaite* (pp. 141–60). Cambridge University Press.

[CR44] Haig B (2016). Tests of statistical significance made sound. Educational and Psychological Measurement.

[CR43] Haig, B. (2020). What can psychology’s statistics reformers learn from the error-statistical perspective? *Methods in Psychology*, *2*, 100020. 10.1016/j.metip.2020.100020

[CR45] Hand, D. J. (1994). Deconstructing statistical questions. *Journal of the Royal Statistical Society, Series A (Statistics in Society)*, *157*(3), 317–356. 10.2307/2983526

[CR47] Hand, D. J. (2014). *The Improbability Principle: Why Coincidences, Miracles, and Rare Events Happen Every Day*. Farrar, Straus, and Giroux.

[CR46] Hand, D. J. (2021). Trustworthiness of statistical inference. *Journal of the Royal Statistical Society: Series A (Statistics in Society)*. 10.1111/rssa.12752.

[CR48] *Harkonen v. United States,* No. 18 (Supreme Court of the United States, filed October 1, 2018). Petition for a Writ of Certiorari. Retrieved December 1, 2020, from https://errorstatistics.files.wordpress.com/2019/06/harkonen-v-us-scotus-2018-petn-cert.pdf.

[CR49] Harrington D, D'Agostino R, Gatsonis C (2019). New guidelines for statistical reporting in the journal. New England Journal of Medicine.

[CR50] Horby, P., Lim, W. S., Emberson, J. R., Mafham, M., Bell, J. L., Linsell, L., Staplin, N., Brightling, C., Ustianowski, A., Elmahi, E., Prudon, B., Green, C., Felton, T., Chadwick, D., Rege, K., Fegan, C., Chappell, L. C., Faust, S. N., Jaki, T., … RECOVERY Collaborative Group. (2021). Dexamethasone in hospitalized patients with covid-19. *The New England Journal of Medicine,**384*(8), 693–704. 10.1056/NEJMoa2021436 10.1056/NEJMoa2021436 PMC7383595 32678530

[CR51] Hurlbert S, Levine R, Utts J (2019). Coup de grâce for a tough old bull: ‘Statistically Significant’ expires. The American Statistician.

[CR52] Ioannidis J (2005). Why most published research findings are false. PLoS Medicine.

[CR53] Ioannidis J (2019). The importance of predefined rules and prespecified statistical analyses: Do not abandon significance. Journal of the American Medical Association (JAMA).

[CR54] Johnson V (2013). Revised standards of statistical evidence. Proceedings of the National Academy of Sciences (PNAS).

[CR55] Kadane, J. (2011). *Principles of uncertainty*. Chapman and Hall/CRC.

[CR56] Kempthorne, O., & Folks, L. (1971). *Probability, statistics, and data analysis* (1st ed.). Iowa State University Press.

[CR57] Lakens D (2019). The value of preregistration for psychological science: A conceptual analysis. Japanese Psychological Review.

[CR58] Lakens D, Adolfi FG, Albers CJ (2018). Justify Your Alpha. Nature Human Behavior.

[CR60] Lehmann E (1993). The Fisher, Neyman–Pearson theories of testing hypotheses: One theory or two?. Journal of the American Statistical Association.

[CR59] Lehmann, E. (2011). *Fisher, Neyman, and the creation of classical statistics* (1st ed.). Springer. 10.1007/978-1-4419-9500-1

[CR61] Lehmann, E., & Romano, J. (2005). *Testing statistical hypotheses* (3rd ed.). Springer.

[CR64] Mayo, D. (1996). *Error and the growth of experimental knowledge*. University of Chicago Press.

[CR65] Mayo, D. (2018). *Statistical inference as severe testing: How to get beyond the statistics wars*. Cambridge University Press. 10.1017/9781107286184

[CR66] Mayo D (2020). P-values on trial: Selective reporting of (best practice guides against) selective reporting. Harvard Data Science Review.

[CR67] Mayo D (2022). The statistics wars and intellectual conflicts of interest (editorial). Conservation Biology.

[CR62] Mayo, D., & Cox, D. (2006). Frequentist statistics as a theory of inductive inference. InJ. Rojo (Ed.), *Optimality: The second Erich L. Lehmann Symposium* (pp. 77–97). Lecture notes—monograph series, 49). Institute of Mathematical Statistics (IMS). 10.1214/074921706000000400

[CR68] Mayo D, Spanos A (2004). Methodology in practice: Statistical misspecification testing. Philosophy of Science.

[CR69] Mayo D, Spanos A (2006). Severe testing as a basic concept in a Neyman–Pearson philosophy of induction. British Journal for the Philosophy of Science.

[CR63] Mayo, D., & Spanos, A. (2011). Error statistics. In P. Bandyopadhyay & M. Forster (Eds.), *Philosophy of statistics* (Vol. 7, pp. 153–198). In D. Gabbay, P. Thagard, & J. Woods (Eds.), *Handbook of philosophy of science*. Elsevier. 10.1016/B978-0-444-51862-0.50005-8

[CR70] McShane BB, Gal D, Gelman A, Robert C, Tackett JL (2019). Abandon statistical significance. American Statistician.

[CR71] Meehl P (1978). Theoretical risks and tabular asterisks: Sir Karl, Sir Ronald, and the slow progress of soft psychology. Journal of Consulting and Clinical Psychology.

[CR72] Morrison, D., & Henkel, R. (Eds.). (1970). *The significance test controversy: A reader*. Aldine De Gruyter.

[CR73] National Academies of Science (NAS). (2019). Consensus study report: Reproducibility and replicability in science. National Academies Press. http://nap.edu/25303. 31596559

[CR74] *NEJM* (*New England Journal of Medicine*). (2019). Author guidelines. Retrieved March 30, 2022, from https://www.nejm.org/author-center/new-manuscripts

[CR76] Neyman, J. (1937). Outline of a theory of statistical estimation based on the classical theory of probability. *Philosophical Transactions of the Royal Society of London, Series A*, *236*(767), 333–380. (Reprinted 1967 in *Early statistical papers of J. Neyman*, 250–290.)

[CR77] Neyman, J. (1957). “Inductive behavior” as a basic concept of philosophy of science. *Revue de l‘Institut International de Statistique/Review of the International Statistical Institute*, *25*(1/3), 7–22. 10.2307/1401671

[CR79] Neyman, J. (1967). *Early statistical papers of J. Neyman*. University of California Press.

[CR80] Neyman J (1976). Tests of statistical hypotheses and their use in studies of natural phenomena. Communications in Statistics: Theory and Methods.

[CR81] Neyman J (1977). Frequentist probability and frequentist statistics. Synthese.

[CR75] Neyman, J., & Pearson, E. (1928). On the use and interpretation of certain test criteria for purposes of statistical inference: Part I. *Biometrika 20A*(1/2), 175–240. 10.2307/2332112 (Reprinted in *Joint statistical papers*, 1–66.)

[CR78] Neyman, J., & Pearson, E. (1967). *Joint statistical papers of J. Neyman and E. S. Pearson*. University of California Press.

[CR82] NISS, National Institute of Statistical Sciences. (2020). The statistics debate! With J. Berger, D. Mayo, and D. Trafimow, moderated by D. Jeske (Link to Video of Debate).

[CR83] Pearson, E., & Neyman, J. (1930). On the problem of two samples. *Bulletin of the Academy of Polish Sciences*, 73–96. (Reprinted 1966 in *Joint statistical papers*, 99–115.)

[CR84] Popper, K. (1959). *The logic of scientific discovery*. Routledge. 10.4324/9780203994627

[CR85] Royall, R. (1997). *Statistical evidence: A likelihood paradigm*. Chapman and Hall, CRC Press. 10.1201/9780203738665

[CR86] Ryan EG, Brock K, Gates S, Slade D (2020). Do we need to adjust for interim analyses in a Bayesian adaptive trial design?. BMC Medical Research Methodology.

[CR87] Selvin, H. (1970). A critique of tests of significance in survey research. In D. Morrison & R. Henkel (Eds.), *The significance test controversy* (pp. 94–106). Aline De Gruyter. 10.4324/9781315134918-14

[CR90] Senn S (2001). Two cheers for P-values?. Journal of Epidemiology and Biostatistics.

[CR89] Senn, S. (2002). A Comment on replication, p-values and evidence, S. N. Goodman. Statistics in Medicine. (1992). 11:875–879. *Statistics in Medicine,**21*(16), 2437–2444. 10.1002/sim.1072 12210627

[CR91] Senn, S. (2007). *Statistical issues in drug development* (2nd ed.). Wiley Interscience.

[CR92] Senn S (2011). You may believe you are a Bayesian but you are probably wrong. Rationality, Markets and Morals (RMM).

[CR88] Senn, S. (2020). A vaccine trial from A to Z with a Postscript (guest post) on *Error Statistics Philosophy Blog.* Retrieved November 12, 2020, from https://errorstatistics.com/2020/11/12/s-senn-a-vaccine-trial-from-a-to-z-with-a-postscript-guest-post/

[CR93] Simmons J, Nelson L, Simonsohn U (2011). False-positive psychology: Undisclosed flexibility in data collection and analysis allow presenting anything as significant. Psychological Science.

[CR94] Sober, E. (2008). *Evidence and evolution: The logic behind the science*. Cambridge University Press.

[CR96] Spanos A (2007). Curve fitting, the reliability of inductive inference, and the error-statistical approach. Philosophy of Science.

[CR97] Spanos A (2010). Is frequentist testing vulnerable to the base-rate fallacy?. Philosophy of Science.

[CR98] Spanos A (2018). Mis-specification testing in retrospect: Mis-specification testing in retrospect. Journal of Economic Surveys.

[CR95] Spanos, A. (2019). Probability theory and statistical inference: Empirical modelling with observational data (2nd ed.). Cambridge University Press. 10.1017/9781316882825

[CR99] Thornton, S., & Xie, M. (2022). Bridging Bayesian, frequentist and fiducial (BFF) inferences using confidence distribution*.* In J. O. Berger, X. L. Meng, N. Reid, & M. Xie (Eds.), *Handbook on Bayesian, Fiducial and Frequentist (BFF) inferences*, Chapman & Hall (forthcoming). https://arxiv.org/abs/2012.04464

[CR101] Wasserstein R, Lazar N (2016). The ASA’s statement on p-values: Context, process and purpose (and supplemental materials). The American Statistician.

[CR100] Wasserstein, R., Schirm, A., & Lazar, N. (2019). Moving to a world beyond “*p* < 0.05” (Editorial). *The American Statistician 73*(S1), 1–19. 10.1080/00031305.2019.1583913

[CR102] Wellek S (2017). A critical evaluation of the current “p-value controversy”. Biometrical Journal/ Biometrische Zeitschrift.

